# Consistent cooling benefits of silvopasture in the tropics

**DOI:** 10.1038/s41467-022-28388-4

**Published:** 2022-02-04

**Authors:** Lucas R. Vargas Zeppetello, Susan C. Cook-Patton, Luke A. Parsons, Nicholas H. Wolff, Timm Kroeger, David S. Battisti, Joseph Bettles, June T. Spector, Arjun Balakumar, Yuta J. Masuda

**Affiliations:** 1 Department of Atmospheric Sciences, University of Washington, Seattle, WA USA; 2 Natural Climate Solutions, The Nature Conservancy, 4245 North Fairfax Drive, Suite 100, Arlington, VA 22203 USA; 3 Nicholas School of the Environment, Duke University, Durham, NC USA; 4 Global Science, The Nature Conservancy, 4245 North Fairfax Drive, Suite 100, Arlington, VA 22203 USA; 5 School of Global Policy and Strategy, University of California San Diego, 9500 Gilman Drive, #0519, La Jolla, CA 92093 USA; 6 Department of Environmental and Occupational Health Sciences, University of Washington, Washington, USA; 7 Stony Brook University Hospital, 101 Nicolls Rd, Stony Brook, NY 11794 USA

**Keywords:** Environmental health, Climate-change mitigation, Climate-change adaptation, Climate-change policy, Sustainability

## Abstract

Agroforestry systems have the potential to sequester carbon and offer numerous benefits to rural communities, but their capacity to offer valuable cooling services has not been quantified on continental scales. Here, we find that trees in pasturelands (“silvopasture”) across Latin America and Africa can offer substantial cooling benefits. These cooling benefits increase linearly by −0.32 °C to −2.4 °C per 10 metric tons of woody carbon per hectare, and importantly do not depend on the spatial extent of the silvopasture systems. Thus, even smallholders can reap important cooling services from intensifying their silvopasture practices. We then map where realistic (but ambitious) silvopasture expansion could counteract a substantial fraction of the local projected warming in 2050 due to climate change. Our findings indicate where and to what extent silvopasture systems can counteract local temperature increases from global climate change and help vulnerable communities adapt to a warming world.

## Abstract (web summary)

A new study shows that tropical silvopasture systems can provide significant cooling services for local communities, and identifies where these silvopasture systems can most effectively counteract global climate change to help communities adapt to warming.

## Introduction

The actions taken in the next decade will be critical for reaching several global milestones, including meeting Nationally Determined Contributions (NDCs) to the Paris Agreement^1^, achieving the Sustainable Development Goals^2^ (SDGs), and restoring ecosystems for people and nature under the United Nations Decade on Restoration^3^. Agroforestry systems (i.e., intentional incorporation of trees and shrubs in agricultural lands) are increasingly seen as one method for simultaneously advancing all three global initiatives^3^. Recent studies estimate that agroforestry systems currently store 6930 Tg C^4^, and that global expansion could sequester up to 284 Tg C/yr^5^. Agroforestry systems can provide significant co-benefits that may be especially important for rural populations in low- and middle-income countries^6^, such as improved soil fertility, more accessible soil water, and increased food security^7–9^. They can also complement efforts to conserve biodiversity^10^ by creating habitat refugia and serving as crucial stepping stones between more intact natural lands^11,12^. In addition to these benefits, a large body of research has quantified many other potential benefits from agroforestry^8,13^ (see also Supplementary Information).

As the climate warms, any environmental change that cools the land surface can provide a socially valuable service, but the extent to which agroforestry can provide cooling benefits is not currently known^8,13,14^. Trees can provide local cooling benefits through shade and evapotranspiration, and the tree’s shade and deep roots can keep surface soil layers that contribute to evapotranspiration moist^15,16^. Cooling benefits are particularly important in tropical regions where many areas already experience temperatures that regularly exceed safety thresholds for outdoor work^17^ and where model projections indicate that the impacts of future warming will be most deleterious^18,19^ and adaptive capacity may be limited^20^. Excess heat reduces labor productivity^21,22^, increases risks of heat stroke^23^, traumatic injuries^24^, and heat-related mortality^18^, and triggers other adverse consequences^25–27^. Potential cooling benefits could be extremely important in tropical rural settings because these regions often lack adequate access to common defenses against heat (e.g., air conditioning, reliable water access).

**Fig. 1:**
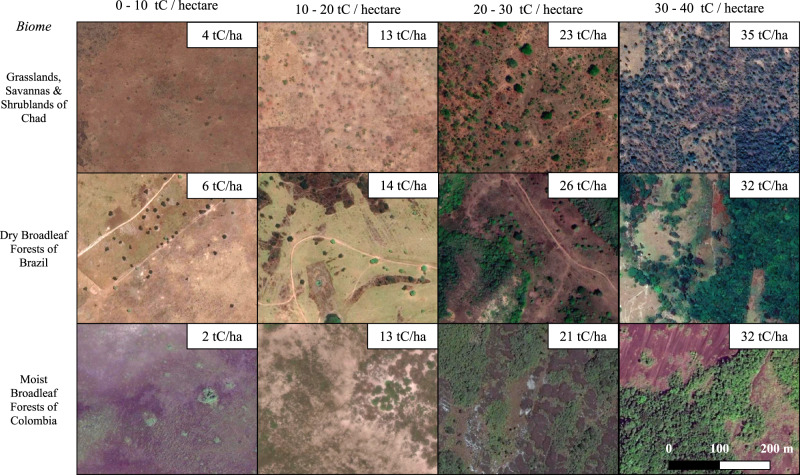
Silvopasture density. A visual guide to silvopasture systems in different biomes and with a range of woody carbon densities. Each row presents a biome type where silvopasture occurs in our study regions in the Americas and Africa, and the columns present a range of carbon density within each biome. Images from Google Satellite and woody carbon density from Chapman et al.^4^. Map data: ©2021 Google.

Here, we examine whether and to what extent the expansion of silvopasture, a kind of agroforestry that involves the deliberate incorporation of trees into pasturelands, could help communities in the tropics better adapt to the negative consequences of heat exposure. We focus specifically on silvopasture for three reasons. First, extreme heat stress is expected to increase in many parts of the tropics and temperate zones not just for people but for all major domesticated animal species^28^, negatively impacting livestock production^29,30^. Second, regulation of temperature for both livestock and people has been identified as a potentially important benefit of silvopasture^14,31^, and third, pasturelands are often targeted by initiatives to expand tree cover^4,32^. We concentrate on the tropics given likely increases in heat exposure in these areas^33^, but limit our analyses to the Americas and Africa because there is relatively little silvopasture in tropical Asia^4,34^. To estimate the cooling benefits of silvopasture, we take advantage of recently published data on woody carbon density in pastureland^4^ (Fig. 1, see Methods) and daytime surface temperature data from the MODIS satellite^35^ (Supplementary Fig. 1).

We answer four questions: (1) What are the cooling benefits of silvopasture across tropical pasturelands in the Americas and Africa? (2) How spatially extensive (contiguous patches of land, or “patch size”) and intensive (amount of woody carbon density per hectare) does silvopasture implementation need to be to achieve cooling benefits? (3) Where are the potential cooling benefits the highest? (4) Finally, how much woody carbon density is required to achieve these cooling benefits?

## Results

### Cooling benefits increase with woody carbon density

**Fig. 2:**
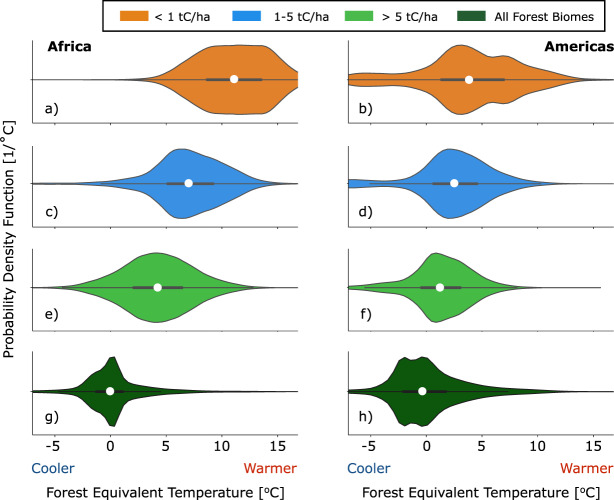
Forest Equivalent Temperature (FET) by silvopasture density. Violin plots of FET in each 1 km^2^ pixel of pasture regions in the Americas and Africa binned by the local woody carbon density in tC/ha, along with a violin plot of FET in each 1 km^2^ pixel of forested regions. In the violin plots, the thick black line in the middle shows the interquartile range and the white circle in the center shows the median value. The probability density functions are smoothed with a Gaussian kernel in each subset of points.

We find that silvopasture systems are cooler, on average, than systems with no or low woody carbon density, and that cooling benefits increase with increasing woody carbon density (Fig. 2). In this study, the cooling benefits of silvopasture are quantified by the forest equivalent temperature (FET) defined as the difference between the temperature at a given location and the temperature of intact tropical forests at the same latitude (see Methods for a further discussion of FET). A FET of 0 ˚C means that the pixel has the same annual mean temperature as intact forests at a given latitude; negative values of FET indicate temperatures lower than the observed average across intact forest at a given latitude, and vice-versa for positive FET values.

On average, the highest FET values (least amount of cooling) are found in pasturelands where woody carbon density is extremely low (less than 1 tC/ha, Fig. 2a, b). We also show the FET of pasturelands where woody carbon density is lower than an established threshold^4^ for what constitutes a silvopasture practice (less than 5 tC/ha, Fig. 2c, d), and where silvopasture practices exist (greater than 5 tC/ha, l Fig. 2e, f). Finally, we show violin plots of FET in intact dry and moist tropical/subtropical broadleaf forests (Fig. 2g, h), which have mean zero by definition (see Methods). The mean values of each subset of points shown in Fig. 2 are significantly different from the others in the study region at the *p* < 0.001 level according to a two-sided t-test.

As shown in Fig. 1, pasturelands with woody carbon density between 1–5 tC/ha have very few trees, yet they exhibit significantly cooler FETs on average compared to pasturelands with low to no woody carbon density (less than 1 tC/ha). The cooling effects of silvopasture systems (i.e., those with woody carbon density greater than 5 tC/ha) are even more pronounced. In general, the frequency distributions and median values shown in Fig. 2 indicate that as the woody carbon density of silvopasture practices increases, the FET decreases and local temperatures converge towards the cooler temperatures found in intact forests (Fig. 2d) within the same latitude.

We next examine how the cooling benefits of silvopasture depend on woody carbon density. In both study regions, the FET of silvopasture decreases linearly with woody carbon density. By taking a linear best-fit across all silvopasture pixels in the study regions, we find that the FET decreases by 1.11 ˚C for every additional 10 tC/ha in the Americas, while in Africa FET of silvopasture decreases by 0.83 ˚C for every additional 10 tC/ha (*p* < 0.001 in both cases). Despite substantial spread in FET (Fig. 2), there is remarkable agreement between the two study regions on the relationship between FET and woody carbon density, suggesting that we are correctly identifying the biophysical impacts of silvopasture with our FET metric.

A comparison with the rate of global warming predicted for 2020 through 2050 in both study regions underscores the importance of the FET-woody carbon density relationship for potentially offsetting local impacts of global climate change. On average, global climate models participating in the Coupled Model Intercomparison Project–Phase 6 (CMIP6) predict that in an accelerating greenhouse gas emissions scenario for the next three decades (through 2050) local warming is 0.56 ˚C/decade in the African study region and 0.51 ˚C/decade in the American study region (see Methods).

### Cooling benefits do not depend on patch size

Larger patches of deforestation are known to increase local temperatures compared to smaller patches^36^, so we hypothesize the converse - that cooling benefits could be amplified by larger silvopasture practices. We use a flood-fill algorithm to quantify the area *A* [km^2^], mean within-patch FET, and mean within-patch woody carbon density of each continuous patch of silvopasture in the study regions (see Methods). We divide the patches into four size classes: small (*A* < 10 km^2^), medium (10 km^2^ < *A* < 33 km^2^), large (33 km^2^ < *A* < 100 km^2^), and extra-large (*A* > 100 km^2^).

**Fig. 3:**
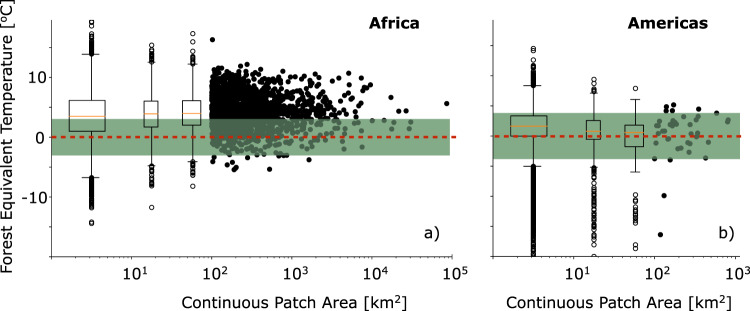
Individual Patch Forest Equivalent Temperature (FET) and continuous patch area. The relationship between contiguous patch area and mean within-patch FET in Africa (left) and the Americas (right). The box plots are aggregates of small, medium, and large patches. The orange lines in the boxes show median values, boxes show the interquartile range, whiskers show the 5^th^–95^th^ percentiles, and open circles show the outliers. Filled black dots show patches larger than 100 km^2^. The green shading shows the standard deviation of the control points (intact forests, see Fig. 2). The red line shows the zero value (a value of zero means that the patch of silvopasture has the same temperature as intact forests at the same latitude).

There is a significant but weak negative correlation between the logarithm of contiguous patch area and mean within-patch FET in the Americas (*r* = −0.07, *p* < 0.001): larger patches of silvopasture tend to be cooler in the American study region (Fig. 3). In Africa, the opposite is true; there is a significant positive correlation between the logarithm of contiguous patch area and mean within-patch FET (*r* = 0.05, *p* < 0.001; see Fig. 3). In both regions, the slope of the best-fit linear regression is weak; an order of magnitude increase in contiguous patch area reduces within-patch FET by only 0.79 ˚C in the Americas, while in Africa, an order of magnitude increase in patch area increases the within-patch FET by 0.29 ˚C. The lack of a strong or consistent relationship between patch size and within-patch FET suggests meaningful cooling benefits are realized for even small contiguous patches of silvopasture.

Mean within-patch woody carbon density is negatively correlated (*p* < 0.001) with FET in all four patch area classes in Africa and in the small and medium patch area classes in the Amazon (Table 1; Supplementary Fig. 2). More importantly, the slopes of the best-fit linear regressions are large in all patch classes; the range in regression values from −1.11 °C per 10tC/ha to −2.84 °C per 10tC/ha suggest that increasing within-patch density, rather than expanding contiguous patch area is the most practical strategy for increasing cooling benefits. The best-fit linear regressions in Table 1 show that in both regions the cooling benefits of silvopasture are not preferentially conferred on the largest patches; indeed in Africa the highest correlation and largest regression slope comes for the smallest patch size class. An important result from this analysis is that in both Africa and the Americas, even small patches of silvopasture can increase the cooling benefits imparted to the landscape by increasing woody carbon density.

**Table 1:** Best-fit linear regression slopes quantifying the relationship between mean within-patch mean Forest Equivalent Temperature (FET) and woody carbon density.

	Small	Medium	Large	X-large
Africa	−2.37 (*r* = −0.35)	−1.90 (*r* = −0.30)	−1.46 (*r* = −0.25)	−1.11 (*r* = −0.20)
Americas	−2.69 (*r* = −0.12)	−2.84 (*r* = −0.13)	NS	NS

Correlations (*r*) between woody carbon density and within-patch mean FET are shown in parentheses. All slopes listed are in °C per 10 tC/ha. All values listed are highly significant at *p* < 0.001, a “NS” denotes a non-significant correlation.

### Cooling benefits in a changing climate - an action map for silvopasture

Currently, silvopasture systems in the Americas and Africa contain 0.20 and 2.55 GtC (billion metric tons of carbon), respectively^4^. To generate cooling equivalent to the projected warming over pasture regions in the decade centered around 2050 relative to the decade centered around 2010 under a high-emissions scenario (see Methods), silvopasture systems would need to add 21 tC of woody carbon per hectare in the Americas and 18 tC per hectare in Africa on average (Supplementary Fig. 3). This land use change throughout the tropics would increase the total pasture woody carbon by a factor of ten in the Amazon and by a factor of four in Africa. This amount of woody carbon could push systems (e.g., native savanna) past their natural state^37^ and/or diminish the productivity of existing grazing lands^38^, so it serves only as an upper bound on additional woody carbon required to completely counteract warming from global climate change.

**Fig. 4:**
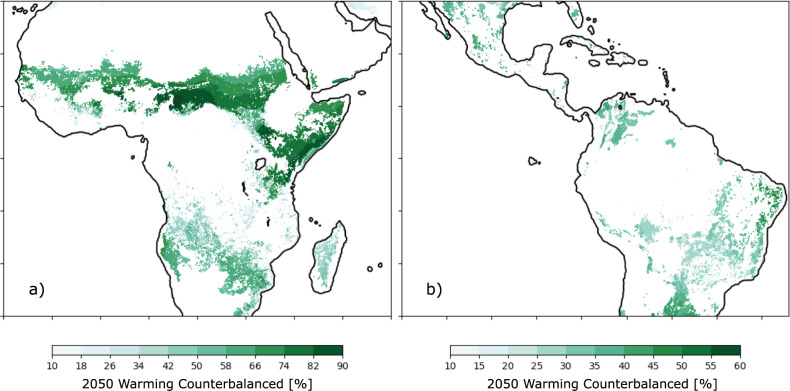
Cooling potential of realistic silvopasture expansion. The percentage of warming expected in 2050 due to anthropogenic emissions that would be counterbalanced in (**a**) Africa and (**b**) the Americas if pasturelands, where woody carbon density is currently less than the biome median value, were increased to their biome median value (see Methods). Coastline data generated from A Global Self-consistent, Hierarchical, High-resolution Geography Database.

A more realistic scenario for silvopasture expansion involves increasing woody carbon density on pasturelands where the density is currently low to an ecologically sustainable value. In Fig. 4 we show the fraction of 2050 warming that could be counterbalanced by increasing woody carbon density to an upper bound set by the median value found in silvopasture systems (woody carbon density > 5 tC/ha) within each biome. Assuming that this threshold value represents an ecologically and economically sustainable amount of woody carbon density, these maps thus identify locations where there is potential for silvopasture to achieve both carbon storage and substantially counteract the projected warming. In other words, these are locations where there is high potential for both climate mitigation and adaptation because woody carbon density is currently below the biome median value. The clearest example of this is the Sahel, where much of the current pastureland has lower woody carbon density than the biome median. Across the Sahel, our results suggest more than 50% of the warming induced by global climate change in 2050 could be counterbalanced by relatively moderate increases in woody carbon density.

Across both study regions, increasing low density silvopasture practices to their biome median value would provide an average cooling benefit of 0.59 °C (0.48 to 0.67 interquartile range) in the Americas and 1.06 °C (0.95 to 1.29 interquartile range) in Africa, as well as store 0.49 GtC of additional carbon in the Amazon and 4.79 GtC in Africa, mostly on pasturelands that currently have very low woody carbon density. We provide estimates of the FET and carbon storage benefits per country (Supplementary Table 2-3) and find that the greatest potential for total carbon storage on pasturelands is in Sudan, Chad, and Somalia.

## Discussion

A recent review of the potential planetary health benefits of agroforestry noted that agroforestry may reduce heat exposure in the rural agricultural areas that already experience adverse heat effects^8^. Importantly, this review found little to no estimates of the current distribution and degree of agroforestry practices that provide these cooling benefits at scale. Our study presents the first comprehensive assessment of the current and potential cooling benefits of silvopasture across the American and African tropics. We find silvopasture practices have the potential to significantly cool the local environment on pasturelands. The best-fit linear regression between woody carbon density and local forest equivalent temperature is −1.11 °C per 10 tC/ha in the Americas and −0.83 °C per 10 tC/ha in Africa. We estimate that a realistic but ambitious silvopasture intensification to biome median woody carbon density could provide 0.59 °C and 1.06 °C of additional cooling, on average, and store a total of 0.49 and 4.79 GtC in silvopasture regions of the Americas and Africa, respectively. Moreover, woody carbon density, not spatial extent, is the biggest determinant of FET on silvopasture lands suggesting that even smallholders could gain the cooling benefits of silvopasture adoption.

Our results mirror previous findings indicating that cooling services provided by trees are substantial in tropical environments^22,36,39–41^. Our findings demonstrate how adding trees to agricultural lands - not just preventing deforestation^39,40,42^ - can decrease local temperature, thus potentially reducing heat exposure of outdoor working populations and livestock in low-latitude countries. Moreover, our analyses are conservative in that they do not capture the additional cooling benefits of shade, which would further minimize heat exposure-related health risks to outdoor workers^24^. Many outdoor workers in rural tropical areas lack basic health protections, and emerging evidence indicates that even basic interventions that include providing shade can reduce the risk of adverse health outcomes such as kidney injury^43^. Hence, agroforestry may be a key component of approaches to reduce heat-related negative health outcomes in rural tropical areas. In addition, cooling services also extend to livestock in these regions, as excess heat loads can decrease productivity, growth, and reproduction, and increase vulnerability to disease^28–30^. The importance of cooling services are increasingly clear given recent work highlighting humid heat impacts on factors such as labor may be underestimated^44^.

That increasing tree cover on human-modified lands, such as pasturelands, can provide cooling benefits is promising, as it demonstrates a shared pathway for advancing human well-being, land conservation or restoration, and climate adaptation and mitigation goals. Several policy efforts are merging to elevate agroforestry for achieving sustainable development and climate change goals. Forty-four out of the 165 countries that have submitted nationally determined contributions for the Paris Climate Agreement mention agroforestry, and 39 of these directly link agroforestry to emissions targets^45^. Agroforestry serves as just one type of natural climate solution^5^, but has great potential in many countries^4^. For human development goals, a recent analysis found agroforestry can contribute to nine of the SDGs^46^, and economic analyses of agroforestry practices suggest agroforestry can be financially profitable for adopters while also generating important offsite benefits^47^. Agroforestry practices have also been identified as a means for avoiding or reducing biodiversity loss on human-modified lands^48^.

Despite the promise of agroforestry, several practical barriers must be overcome to scale up its implementation. Policies must be carefully designed to avoid known pitfalls in agroforestry policy implementation^13,49^. Recent large-scale analyses identifying ideal areas for increasing tree cover indicate that careful selection of areas conducive to such efforts is important^50^. Particular caution is merited in systems that naturally support low tree densities (e.g., xeric and montane shrublands and grasslands)^51^, which we excluded from our analysis (see Methods). Although trees and agroforestry systems can be found in these locations (e.g., parklands in the Sahel), trees can negatively impact grassland biodiversity^52,53^ so we conservatively excluded these from our analysis to avoid suggesting perverse biodiversity consequences. Adding trees to crop-only systems can result in trade-offs with crop yields^54,55^ and in some cases negative social and environmental impacts^56^, which can be minimized or avoided through appropriate species selection^54,57^ and more inclusive planning process that account for the needs of the local communities^56^. Land-use histories must also be considered when targeting areas for agroforestry, as, for instance, biodiversity benefits from agroforestry result from planting trees in existing agricultural lands rather than converting ‘pristine’ habitats or converting forests to agroforestry^58^. Similarly, site-level assessments are necessary to determine the tree (or shrub) density and species for optimizing grazing productivity and carbon storage^37^.

Incentives and support for landholders must also be improved. Many rural populations in low- and middle-income countries with weak institutions face significant tenure insecurity^59^, thus reducing incentives to invest in long-term land management practices^60,61^. Both state and non-state actors can reduce barriers to agroforestry adoption^62^, such as by facilitating access to credit and markets, building farmer knowledge and capacity, and creating sustainable supply chains. Several policy guides exist to help policymakers overcome these barriers^49,63^.

While our study advances the understanding of the cooling benefits of agroforestry, it has several limitations. Ideally, we would know the initiation date of the silviculture practice; since this information is not available, we used above-ground carbon density to assess the cooling benefits of silviculture rather than investigating the temperature change before and after the practice began. We limited our analysis to pasturelands, so it is unclear whether the benefits for croplands would differ. Our analytic framework focuses on carbon stocks rather than specific silvopasture practices (e.g., arrangement of trees) or tree species, which provides a practical but grounded analytic approach for estimating cooling benefits at large scales. However, future work should examine whether specific tree spatial arrangements or species yield varying cooling benefits, as has been documented for urban trees^64^. Moreover, we only focused on aboveground carbon stocks which drive temperature differences, but additional carbon accumulation can also occur in belowground and soil pools^7^. Finally, the satellite data provides information on the canopy surface temperature in regions where tree cover is dense. As such, our study uses our forest equivalent temperature metric as a proxy for ground-level temperature reduction, a key determinant of human^60^ and livestock thermal comfort^61^. Field-level studies suggest the cooling benefits are substantial, with one study in Ethiopia finding areas with agroforestry were up to 6 ˚C cooler compared to open fields^65^, but more field work is needed to verify the results presented here on a large scale. Field studies and high-resolution data would provide more confidence in the results presented here which are limited in scale by the spatial resolution of the temperature data (>1 km^2^).

As momentum builds for increasing the density of trees on agricultural lands in low- and middle-income countries, there is a need to fully articulate and understand the potential co-benefits trees provide to rural communities^8,13^. For national governments and others making investments in such efforts for global biodiversity, climate change, and SDGs, understanding where agroforestry can simultaneously address multiple goals is essential, but any effort must be balanced by grounded assessments of restoration and tree planting potential. Our results can be used with data on where agroforestry programs can increase food security, carbon storage, and other outcomes of interest to target efforts to expand agroforestry to benefit local populations.

## Methods

### Data sources

Our analysis requires information on both woody carbon density and temperature throughout the tropics on a scale fine enough to distinguish places where smallholder silvopasture could impart cooling benefits. For woody carbon density, we used global aboveground woody biomass density data from Chapman et al.^4^, which partitions information for agroforestry on specifically defined pasture and agricultural lands. While no extensive and comprehensive maps of silvopasture practices exist, we used the spatial layer from Chapman et al.^4^ that maps aboveground biomass density within known pasturelands as a proxy for silvopasture. These data were regridded from 30 m^2^ to 1 km^2^ (the resolution of the temperature data) by multiplying the mean biomass density on pastureland within each 1 km^2^ square by the fraction of each square occupied by pastureland. Each 1 km^2^ grid is referred to as a “pixel” in this study. To prevent contamination by pixels with very sparse silvopasture practices, we only included pixels where more than half of the 1 km^2^ area is covered by silvopasture in our analysis. To convert from aboveground woody biomass density to wood carbon density, we multiplied the data in the Chapman et al.^4^ layer by 0.47.

Planting trees in native grasslands can have adverse biodiversity consequences^66^ and these trees have experienced high mortality rates^67^. We, therefore, used biome designations from Dinerstein et al.^68^ to remove silvopasture from our analysis that is located on montane grass and shrublands, along with deserts and xeric shrublands where silvopasture is unlikely to be viable due to water and nutrient limitations (this reduces total agroforestry by 16% in the Americas and 6% in Africa). Finally, we excluded mangrove biomes because they are highly dynamic systems that require complex accounting for in situ versus exported carbon^69^. We show maps of woody carbon density in terms of tons C per hectare (tC/ha) for silvopasture systems within the remaining areas of the two study regions in Supplementary Fig. 1a, b.

Temperature data come from the MODIS Aqua satellite^35^, which provides daytime (1:30 PM local time) surface temperatures across the globe. We use annual averaged daytime temperatures from the year 2018, a year in which the El Niño Southern Oscillation was relatively inactive, allowing for a more accurate estimation of the biophysical impacts of silvopasture on local temperatures that do not include the potential external influence of ocean temperatures. Since temperatures are highest and the influence of vegetation on turbulent energy fluxes is greatest in the middle of the day, the MODIS Aqua observations provide the best temperature observational dataset for a study aimed at understanding how silvopasture impacts local temperatures. The MODIS data are available at 1 km^2^ resolution and are not regridded in this analysis. Maps of annual mean daytime surface temperature for the two study regions are shown in Supplementary Fig. 1c, d.

We calculated the projected warming for 2050 using output from 24 global climate models participating in CIMP6 (models listed in Supplementary Table 1) forced by the SSP5-8.5 scenario of human activity (land use) and emissions, which shows a median global averaged warming of 1.25 °C compared to the global average temperature in the decade centered on 2010. Although SSP5-8.5 is a high-end emission scenario, the projected warming over this 30-year horizon using a low-end emission scenario (SSP1-2.6) is 0.75 °C^70,71^. This difference is roughly equivalent (60%) to the fraction of warming that is avoided by silvopasture (~ 30–80%; Fig. 4), meaning that in a warming scenario less severe than the one we present in our analysis, adding silvopasture could counteract a higher fraction (or potentially all) of the local anthropogenic warming in 2050. The data and code for this study has been deposited at 10.5281/zenodo.5787022.

### Definition of forest equivalent temperature

The ideal data set to quantify the cooling due to silvopasture would indicate the date that silvopasture was implemented for each pixel in the tropics; in this case, a difference in the averaged temperature before and after implementation would give a precise measure of the cooling induced by additional woody carbon density because averaging would remove the unrelated year-to-year natural variability. However, the Chapman et al.^4^ data set does not indicate implementation date; rather those authors indicate (for a range of years) the pixels with silvopasture. Without temporal information, we defined the forest equivalent temperature (FET) as the difference between the average annual mean temperature at the pixel in question, and the average annual average temperature of the intact forests at the latitude of the pixel in question. We calculated the FET using the annual temperature at each pixel for one year (2018) and stratified the results based on woody carbon density for each pixel (see Fig. 2). We then subtracted this temperature from the average temperature of intact forests at the same latitude in 2018 to arrive at FET. The use of a single year introduces noise associated with natural temperature variability (regional in spatial extent), but not so much as to obscure the relationship between silvopasture intensity and FET. The set of pixels that qualify as “intact forests” are those classified by Dinerstein et al.^68^ as “tropical and subtropical dry and moist broadleaf forests”—these regions are most ecologically similar to high-density silvopasture practices. The latitudinal mean approach was used in this study to eliminate the continental scale changes in mean solar radiation found across our study region.

Very little silvopasture is actually practiced on tropical/subtropical moist and dry broadleaf forests, and we have included Fig. 1 as a visual guide to woody carbon density to show how the most intense silvopasture practices may approach actual forest biomes. We experimented with different sets of control points (rather than intact forests), including the latitudinal mean of low carbon pasturelands, tropical and subtropical grasslands, and all biomes, but found that the spread in tropical/subtropical dry and moist broadleaf forests was the smallest of all sets we examined. The set of intact forest points best fits our need for a homogenous dataset of control points, likely because the high evapotranspiration and available soil moisture in these forested regions limits temperature variability in response to solar radiation and precipitation fluctuations that create larger temperature fluctuations in more arid regions.

The MODIS satellite measures some mixture of canopy and surface temperature in regions where agroforestry is practiced rather than the two-meter air temperature that is a common metric of heat exposure. Here we are attempting to illuminate the potential cooling services offered by increasing the woody carbon density of silvopasture by comparing canopy temperatures across space. However, temperatures beneath tropical tree canopy are lower than canopy surface temperatures^72^ due to the canopy’s shading effect. Since the impact of canopy shading on beneath canopy temperatures likely scales with woody carbon density, we believe our estimate of FET to be a conservative estimate of on-the-ground cooling.

### Analysis of forest equivalent temperature

To generate Fig. 2, we composited all 1 km^2^ pixels within the study regions by their woody carbon density (or biome in the case of the intact forest violin plots in Fig. 2g, h). We used a linear best fit to quantify the relationship between woody carbon density and FET in both study regions. To further explore the relationship between woody carbon density and FET, we used a flood-fill algorithm to isolate continuous patches of silvopasture and quantify the average FET and woody carbon density within them. This algorithm iterates through the Chapman et al.^4^ dataset to find silvopasture pixels that share at least one boundary. While this method allows us to find continuous patches of silvopasture and record the variables of interest (woody carbon density and FET) within them, it does not discriminate between patches with different shapes; a long thin line of silvopasture pixels agroforestry will be counted identically to a perfect circle of pixels with the same area. Nevertheless, this method allows a precise quantification of the biophysical impacts of agroforestry in a way that large-scale satellite averaging does not.

### Sources of uncertainty in our analysis

One important source of uncertainty in our analysis is that we have only looked at one year of data-2018. While this is a necessary compromise with the period of the Chapman et al.^4^ data set, interannual climate variability creates some unavoidable noise that is reflected in the spread of FET values shown in Figs. 2, 3. We expect interannual climate variability in this year, when the El Nino Southern Oscillation was relatively neutral, to be normally distributed with no warm/cold bias in respect to the longer climate record.

While we have removed the latitudinal variability in solar radiation by taking the average of temperatures in forest biomes as our control points, there are zonal inhomogeneities that influence temperature across both study regions, and particularly in Africa (see Supplementary Fig. 1). Importantly, because forests are cooler than all other major biomes in the tropics on average, the uncertainty induced by zonal inhomogeneities should skew towards warmer values. Despite this potential bias, we still recover the cooling signal from agroforestry in our analysis, though zonal inhomogeneities undoubtedly contribute some noise along with interannual climate variability.

Finally, there is inherent uncertainty in temperature and aboveground woody carbon density, which are derived from satellite products. However, we are not aware of errors in these products that would bias our results and thus do not view them as a major concern for our analysis.

### Creating the action maps

To create the maps shown in Fig. 3, we used the linear equation:

1 $$\varDelta T=m\varDelta C.$$

In Eq. 1, $\varDelta T$is the FET change driven by changing woody carbon density$\varDelta C$, and *m* is the slope of the best-fit linear regression between the two quantities obtained from the individual pixel analysis (*m* = −1.11 °C per 10 tC/ha in the Americas, *m* = −0.83 °C per 10 tC/ha in Africa) to make the most conservative estimate that does not account for the possible cooling benefits associated with larger contiguous patches of silvopasture. Using the CMIP6 model ensemble (Supplementary Table 1), we calculated local temperature change over the two study regions between the climatological average temperatures from 2045–2055 and from 2015–2025. CMIP6 model output was downloaded from the Earth System Federation grid and regridded to a common spacing equivalent to the MODIS grid for better comparison with all other data sets. By dividing this temperature change by the slope *m*, we estimate the additional woody carbon density that, if added to each grid cell, would provide local cooling equivalent to the warming projected by climate models roughly 30 years from now under a high emission scenario (e.g., SSP5-8.5) shown in Supplementary Fig. 4. Low emission scenarios (e.g., RCP 4.5) warm by ~ 60% of the high emission scenarios^73^.

In some regions, the amount of woody carbon density required to counteract the warming is not feasible, either because of biological limitations of the regions (i.e., negative impacts on grassland biodiversity, insufficient water, sunlight, soil nutrients, etc.) or because these regions are already under high density silvopasture practices, thus making adding more trees impractical. Therefore, to make Fig. 3, we calculate $\varDelta C$ by assuming that only places where current woody carbon density on pasturelands is less than the median value of silvopasture (>5 tC/ha) on their respective biomes can increase. By assuming that all these pixels (where current woody carbon density is less than the biome median silvopasture value) increase to their respective biome median values, we use Eq. 1 to compute the change in temperature attributable to increasing woody carbon density to this ecologically reasonable goal. By dividing this temperature change by the global warming signal from the CMIP6 models, we arrive at the percentage values shown in Fig. 3.

## Supplementary information

Supplementary Information

## Data Availability

The data for this study has been deposited at 10.5281/zenodo.5787022.

## Code availability

Code used for analyses are available at 10.5281/zenodo.5787022.
