# Supplementary material for: Consistent cooling benefits of silvopasture in the tropics
Source: Nat Commun. 2022 Feb 4;13:708. doi: 10.1038/s41467-022-28388-4 (PMC8816911; doi:10.1038/s41467-022-28388-4)
Supplement: Supplementary file 1 — Supplementary Information [file 41467_2022_28388_MOESM1_ESM.docx]

**Supplementary Information for Consistent cooling benefits of silvopasture in the tropics**

**Other health co-benefits of agroforestry systems**

Our study highlights the cooling services provided by agroforestry systems on pasturelands, but a large body of evidence has demonstrated agroforestry systems can provide other health co-benefits, highlighting how these systems can be an asset for other public health initiatives. In general, decreased heat exposure via local cooling benefits increases productivity, decreases exacerbation of chronic disease, and improves mental health outcomes^1,2^. Increasing forest cover increases native air-filtering systems that can improve air quality, resulting in decreased exacerbations of chronic lung diseases such as asthma and COPD^3^. Agroforestry systems also provide increased nutrition security by providing a stable, climate-resistant source of healthy foods^4^. Access to a consistent source of healthy foods helps communities located in historical food deserts overcome traditional barriers to a healthier diet, which in turn reduces risk of obesity, diabetes, and other cardiovascular disease. By changing current land use rather than increasing land conversion for agricultural use, there is a decreased chance of interaction with emerging, novel human pathogens. By providing alternate, more stable and environmentally friendly sources of income, there is greater healthcare affordability. Agroforestry can also provide sources of naturally-grown medicine, without reducing yields for cash crops required for income^5,6^.

**
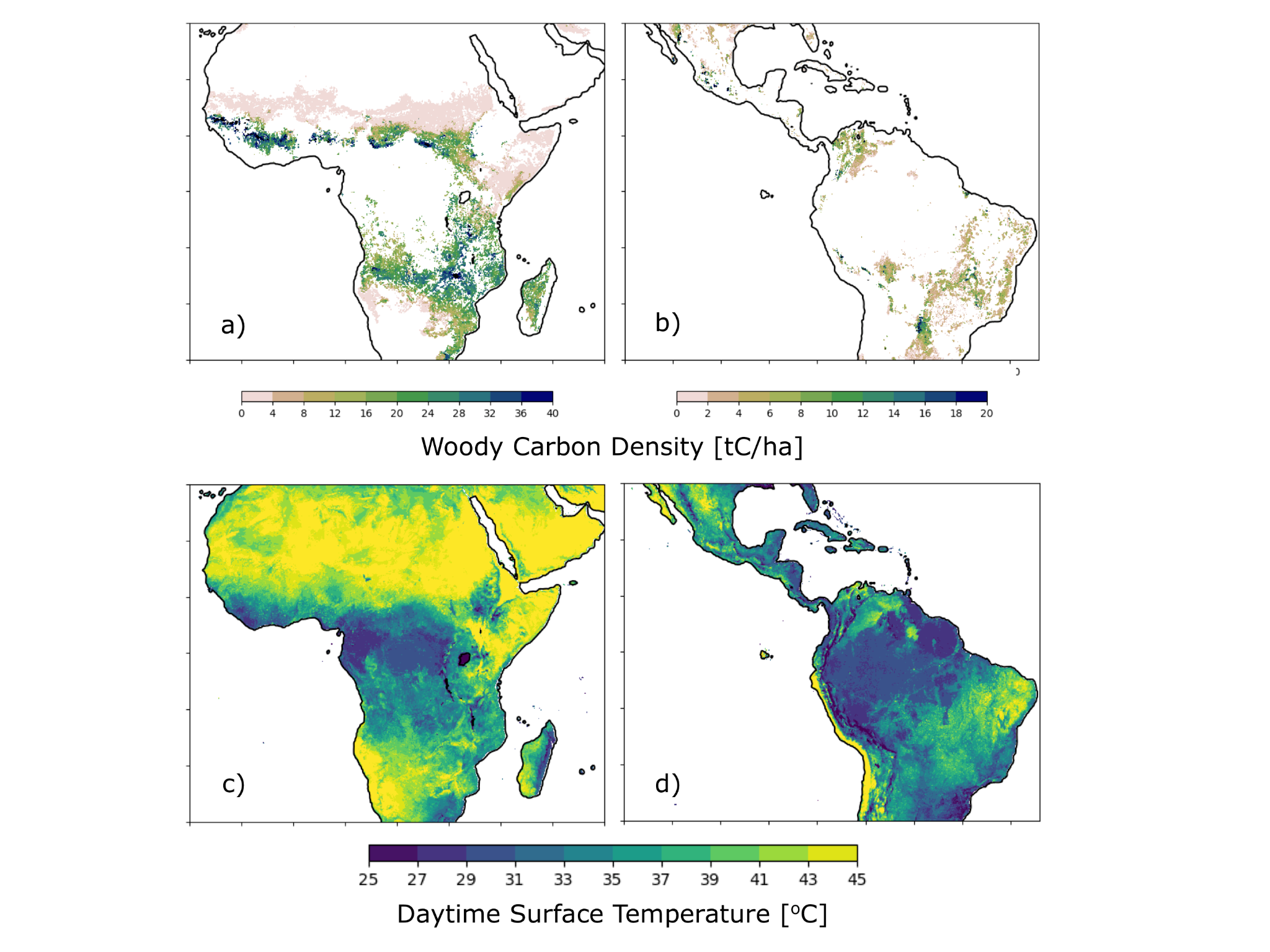
**

**Supplementary Figure 1 Silvopasture density and Daytime Temperatures:** Woody carbon density from the Chapman et al^7^ dataset for Africa (a) and the Americas (b). We have removed carbon density data from ecosystems dominated by montane grass and shrublands, deserts and xeric shrublands, and mangrove biomes. Daytime surface temperature from the MODIS satellite averaged over all months in 2018 in Africa (c) and the Americas (d). Coastline data generated from A Global Self-consistent, Hierarchical, High-resolution Geography Database.


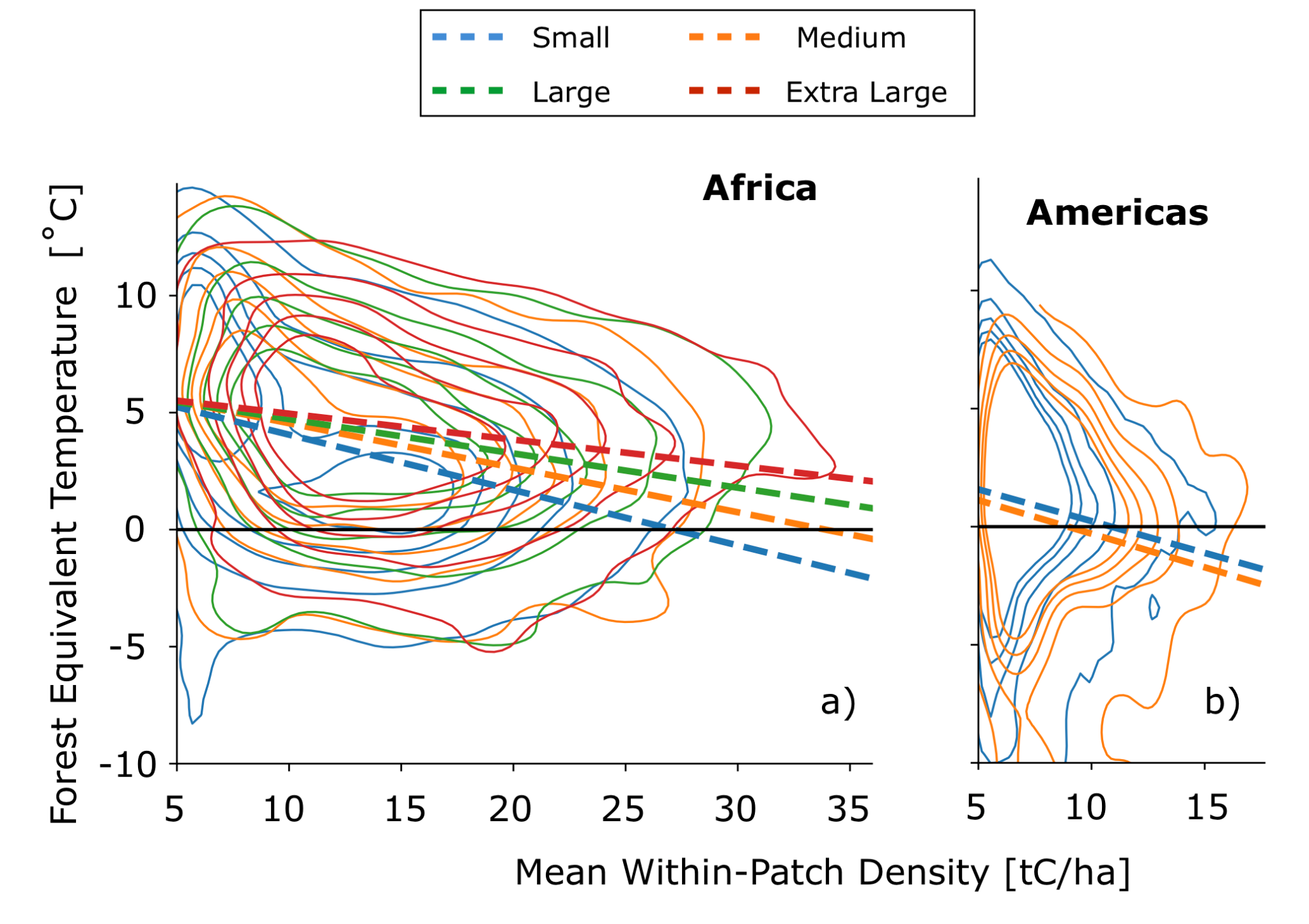


**Supplementary Figure 2 Silvopasture Patch Density and FET:** Contour plots showing the relationship between mean within-patch woody carbon density and mean within-patch FET in the four patch size classes of silvopasture across Africa (a) and the Americas (b). The dashed lines show linear best-fit regressions for each size composite. The black line shows the zero value for FET (a value of zero means that the patch of silvopasture has the same temperature as forests at the same latitude). We only show contours and regressions for patch size classes where the correlation between mean within-patch density and FET is statistically significant (see Table 1).

**
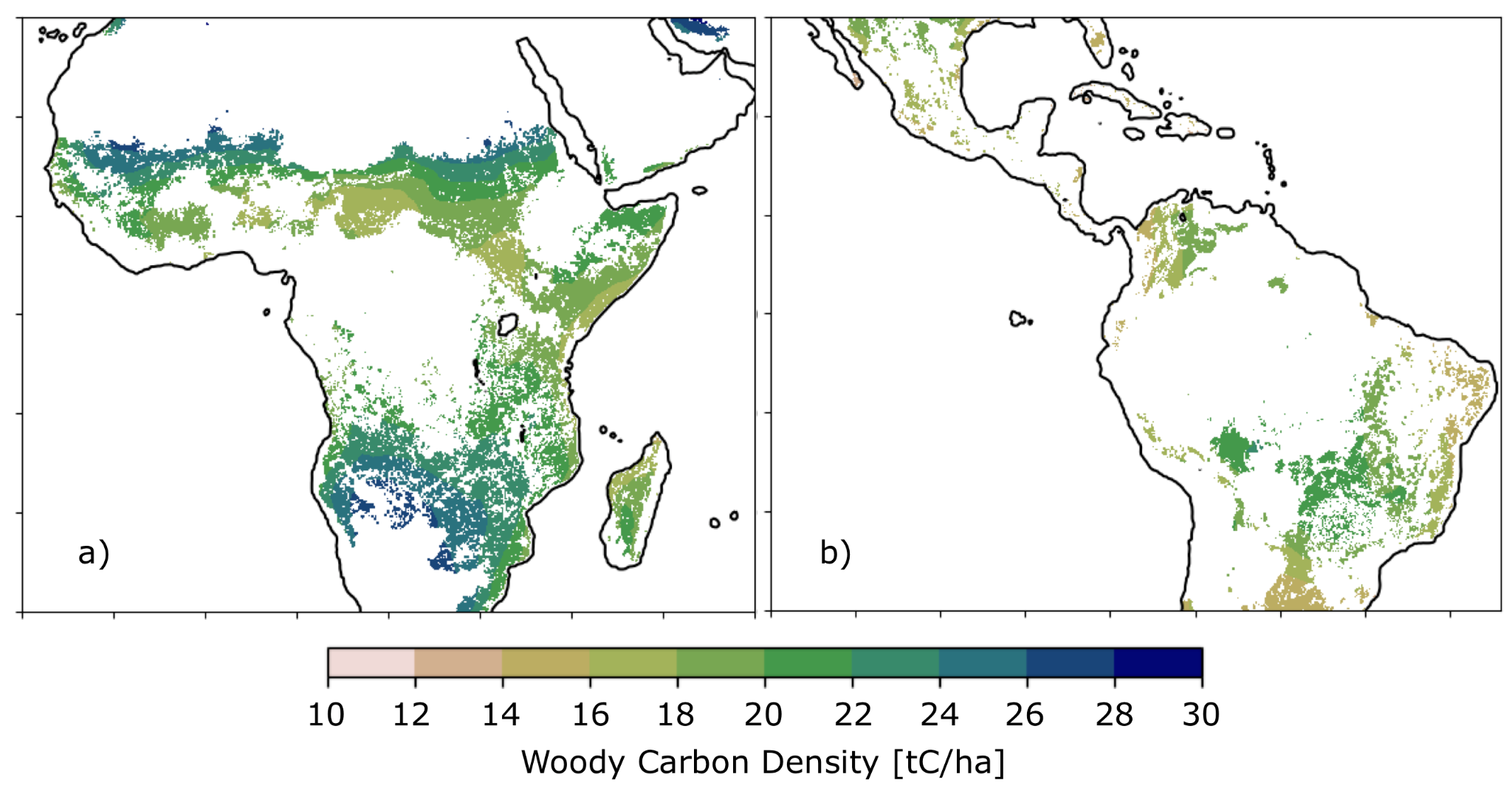
**

**Supplementary Figure 3 Biomass Required to Counterbalance Local Warming:** Additional woody carbon density required in Africa (a) and the Americas (b) to fully counterbalance local warming on pasturelands in 2050. Coastline data generated from A Global Self-consistent, Hierarchical, High-resolution Geography Database.

**Supplementary Table 1:** CMIP6 models analyzed

| **Model Name** |
| --- |
| ACCESS-CM2 |
| ACCESS-ESM1-5 |
| CanESM5 |
| CESM2 |
| CESM2-WACCM |
| CNRM-CM6-1 |
| CNRM-CM5-1-HR |
| CNRM-ESM2-1 |
| EC-Earth3 |
| EC-Earth3-Veg |
| GFDL-ESM4 |
| GISS-E2-1-G |
| HadGEM3-GC31-LL |
| INM-CM4-8 |
| INM-CM5-0 |
| IPSL-CM6A-LF |
| MIROC6 |
| MIROC-ES2L |
| MPI-ESM1-2-HR |
| MPI-ESM1-2-LR |
| MRI-ESM2-0 |
| NorESM2-MM |
| NorESM2-LM |
| UKESM1-0-LL |

**Supplementary Table 2:** Viable woody carbon density on pasturelands from agroforestry and associated projected cooling in the Americas.

|  |  |  | **Viable biomass** | | | | | **Cooling** | | | |
| --- | --- | --- | --- | --- | --- | --- | --- | --- | --- | --- | --- |
|  | Cell count | Hectares | Total tC | Mean tC/ha | Median tC/ha | 25% qrtl tC/ha | 75% qrtl tC/ha | Mean˚C | Median ˚C | 25% qrtl ˚C | 75% qrtl ˚C |
| Argentina | 100,062 | 7,570,312 | 39,705,016 | 5.25 | 4.98 | 5.43 | 5.98 | 0.33 | 0.31 | 0.34 | 0.37 |
| Bolivia | 117,301 | 9,748,726 | 51,702,140 | 5.30 | 4.89 | 5.72 | 6.10 | 0.33 | 0.30 | 0.35 | 0.38 |
| Brazil | 355,815 | 29,438,978 | 139,428,496 | 4.73 | 3.97 | 4.95 | 5.76 | 0.29 | 0.25 | 0.31 | 0.36 |
| Chile | 11,643 | 870,417 | 4,532,320 | 5.21 | 5.23 | 5.24 | 5.24 | 0.32 | 0.32 | 0.33 | 0.33 |
| Colombia | 118,182 | 10,108,965 | 50,972,060 | 5.04 | 4.53 | 5.59 | 5.98 | 0.31 | 0.28 | 0.35 | 0.37 |
| Costa Rica | 324 | 27,438 | 122,128 | 4.45 | 3.92 | 4.68 | 5.32 | 0.28 | 0.24 | 0.29 | 0.33 |
| Cuba | 950 | 75,992 | 399,172 | 5.26 | 4.60 | 5.60 | 6.29 | 0.33 | 0.29 | 0.35 | 0.39 |
| Dominican Republic | 304 | 24,770 | 127,738 | 5.16 | 4.49 | 5.62 | 6.31 | 0.32 | 0.28 | 0.35 | 0.39 |
| Ecuador | 997 | 85,770 | 298,116 | 3.48 | 2.01 | 3.64 | 5.03 | 0.22 | 0.12 | 0.23 | 0.31 |
| El Salvador | 42 | 3,511 | 12,982 | 3.70 | 2.30 | 3.74 | 5.29 | 0.23 | 0.14 | 0.23 | 0.33 |
| Guatemala | 135 | 11,173 | 38,557 | 3.45 | 2.75 | 3.64 | 4.14 | 0.21 | 0.17 | 0.23 | 0.26 |
| Guyana | 6,703 | 576,219 | 3,439,370 | 5.97 | 5.91 | 6.07 | 6.15 | 0.37 | 0.37 | 0.38 | 0.38 |
| Haiti | 9 | 731 | 3,973 | 5.43 | 5.11 | 5.52 | 5.67 | 0.34 | 0.32 | 0.34 | 0.35 |
| Honduras | 3 | 251 | 731 | 2.91 | 0.93 | 2.71 | 4.95 | 0.18 | 0.06 | 0.17 | 0.31 |
| Mexico | 91,422 | 7,012,642 | 38,517,504 | 5.50 | 4.84 | 6.13 | 6.73 | 0.34 | 0.30 | 0.38 | 0.42 |
| Nicaragua | 3,618 | 301,951 | 1,192,048 | 3.95 | 3.06 | 4.22 | 5.10 | 0.24 | 0.19 | 0.26 | 0.32 |
| Panama | 7 | 596 | 2,092 | 3.51 | 3.01 | 3.58 | 4.27 | 0.22 | 0.19 | 0.22 | 0.26 |
| Paraguay | 58,853 | 4,583,240 | 18,873,734 | 4.12 | 3.15 | 4.44 | 5.36 | 0.26 | 0.20 | 0.28 | 0.33 |
| Peru | 13,009 | 1,089,295 | 5,228,438 | 4.80 | 4.17 | 5.61 | 5.86 | 0.30 | 0.26 | 0.35 | 0.36 |
| USA | 50,467 | 3,808,571 | 18,735,112 | 4.91 | 4.35 | 4.90 | 5.95 | 0.30 | 0.27 | 0.30 | 0.37 |
| Venezuela | 56,497 | 4,814,548 | 24,136,170 | 5.01 | 4.27 | 5.64 | 6.09 | 0.31 | 0.26 | 0.35 | 0.38 |

**Supplementary Table 3:** Viable woody carbon density on pasturelands from agroforestry and associated projected cooling to counteract 2050 average warming for countries in Africa.

|  |  |  | **Viable biomass** | | | | | **Cooling** | | | |
| --- | --- | --- | --- | --- | --- | --- | --- | --- | --- | --- | --- |
|  | Cell count | Hectares | Total tC | Mean tC/ha | Median tC/ha | 25% qrtl tC/ha | 75% qrtl tC/ha | Mean˚C | Median ˚C | 25% qrtl ˚C | 75% qrtl ˚C |
| Algeria | 234 | 18,085 | 238,264 | 13.12 | 11.73 | 11.79 | 14.97 | 1.13 | 1.01 | 1.01 | 1.29 |
| Sudan | 704,216 | 58,860,703 | 862,139,456 | 14.65 | 14.97 | 14.97 | 14.97 | 1.26 | 1.29 | 1.29 | 1.29 |
| Angola | 159,455 | 13,304,798 | 88,884,088 | 6.70 | 2.95 | 5.75 | 10.12 | 0.58 | 0.25 | 0.49 | 0.87 |
| Benin | 133 | 11,192 | 150,308 | 13.44 | 13.84 | 14.49 | 14.55 | 1.16 | 1.19 | 1.25 | 1.25 |
| Botswana | 118,961 | 9,524,995 | 127,440,200 | 13.39 | 11.85 | 14.80 | 14.97 | 1.15 | 1.02 | 1.27 | 1.29 |
| Burkina Faso | 14,328 | 1,204,616 | 14,464,056 | 12.03 | 9.20 | 14.97 | 14.98 | 1.03 | 0.79 | 1.29 | 1.29 |
| Burundi | 282 | 24,224 | 45,404 | 1.87 | 0.54 | 1.40 | 2.53 | 0.16 | 0.05 | 0.12 | 0.22 |
| Cameroon | 3,627 | 305,396 | 3,983,671 | 13.04 | 11.73 | 13.92 | 14.97 | 1.12 | 1.01 | 1.20 | 1.29 |
| CAR | 3,398 | 287,406 | 3,767,258 | 13.11 | 13.40 | 14.75 | 14.95 | 1.13 | 1.15 | 1.27 | 1.29 |
| Chad | 408,159 | 34,235,532 | 465,366,592 | 13.61 | 14.29 | 14.97 | 14.97 | 1.17 | 1.23 | 1.29 | 1.29 |
| Congo | 1,977 | 170,015 | 770,198 | 4.53 | 1.69 | 3.74 | 6.67 | 0.39 | 0.15 | 0.32 | 0.57 |
| Côte d’Ivoire | 13,359 | 1,135,632 | 3,097,488 | 2.73 | 0.92 | 2.10 | 3.85 | 0.23 | 0.08 | 0.18 | 0.33 |
| DRC | 4,517 | 385,343 | 1,637,300 | 4.25 | 2.03 | 3.97 | 6.15 | 0.37 | 0.17 | 0.34 | 0.53 |
| South Sudan | 210,014 | 17,896,939 | 168,544,448 | 9.42 | 5.43 | 10.44 | 14.23 | 0.81 | 0.47 | 0.90 | 1.22 |
| Eritrea | 49,679 | 4,114,199 | 61,452,224 | 14.94 | 14.97 | 14.97 | 14.97 | 1.28 | 1.29 | 1.29 | 1.29 |
| Ethiopia | 95,583 | 8,171,446 | 119,315,904 | 14.60 | 14.95 | 14.97 | 14.97 | 1.26 | 1.29 | 1.29 | 1.29 |
| Gabon | 2,035 | 175,130 | 1,334,177 | 7.62 | 4.28 | 7.89 | 11.31 | 0.66 | 0.37 | 0.68 | 0.97 |
| Gambia | 2,512 | 210,178 | 1,781,076 | 8.47 | 4.49 | 8.79 | 12.84 | 0.73 | 0.39 | 0.76 | 1.10 |
| Ghana | 31,711 | 2,692,393 | 15,025,451 | 5.59 | 2.01 | 4.59 | 8.38 | 0.48 | 0.17 | 0.39 | 0.72 |
| Abyei | 4,828 | 409,359 | 3,618,137 | 8.84 | 5.21 | 9.20 | 12.81 | 0.76 | 0.45 | 0.79 | 1.10 |
| Guinea-Bissau | 498 | 41,914 | 109,485 | 2.61 | 0.93 | 2.14 | 3.88 | 0.22 | 0.08 | 0.18 | 0.33 |
| Guinea | 2,131 | 180,140 | 642,460 | 3.57 | 1.10 | 2.61 | 5.24 | 0.31 | 0.09 | 0.22 | 0.45 |
| Iran | 60,640 | 4,557,495 | 29,683,890 | 6.51 | 6.50 | 6.50 | 6.50 | 0.56 | 0.56 | 0.56 | 0.56 |
| Kenya | 229,618 | 19,748,841 | 287,932,064 | 14.58 | 14.96 | 14.97 | 14.97 | 1.25 | 1.29 | 1.29 | 1.29 |
| Lesotho | 12,633 | 945,875 | 10,919,983 | 11.55 | 10.51 | 12.12 | 13.21 | 0.99 | 0.90 | 1.04 | 1.14 |
| Madagascar | 113,960 | 9,247,635 | 43,178,252 | 4.68 | 2.57 | 4.68 | 6.72 | 0.40 | 0.22 | 0.40 | 0.58 |
| Malawi | 598 | 49,935 | 95,306 | 1.91 | 0.53 | 1.23 | 2.18 | 0.16 | 0.05 | 0.11 | 0.19 |
| Mali | 250,166 | 20,739,241 | 298,448,640 | 14.40 | 14.97 | 14.97 | 14.97 | 1.24 | 1.29 | 1.29 | 1.29 |
| Mauritania | 115,032 | 9,491,262 | 142,090,272 | 14.97 | 14.97 | 14.97 | 14.97 | 1.29 | 1.29 | 1.29 | 1.29 |
| Morocco | 15,110 | 1,133,128 | 5,828,825 | 5.14 | 5.16 | 5.16 | 5.16 | 0.44 | 0.44 | 0.44 | 0.44 |
| Mozambique | 92,609 | 7,371,883 | 39,062,116 | 5.31 | 2.34 | 4.64 | 7.61 | 0.46 | 0.20 | 0.40 | 0.65 |
| Namibia | 122,193 | 9,917,638 | 141,673,664 | 14.29 | 14.90 | 14.97 | 14.97 | 1.23 | 1.28 | 1.29 | 1.29 |
| Niger | 206,450 | 17,121,632 | 255,928,976 | 14.95 | 14.97 | 14.97 | 14.97 | 1.29 | 1.29 | 1.29 | 1.29 |
| Nigeria | 52,055 | 4,391,795 | 54,054,060 | 12.32 | 11.34 | 14.63 | 14.97 | 1.06 | 0.98 | 1.26 | 1.29 |
| Oman | 104 | 8,556 | 128,521 | 15.02 | 14.97 | 14.97 | 15.08 | 1.29 | 1.29 | 1.29 | 1.30 |
| Rwanda | 720 | 61,940 | 240,941 | 3.89 | 1.35 | 2.81 | 5.30 | 0.33 | 0.12 | 0.24 | 0.46 |
| Senegal | 20,697 | 1,719,845 | 24,791,320 | 14.42 | 14.94 | 14.97 | 14.97 | 1.24 | 1.28 | 1.29 | 1.29 |
| Somalia | 375,734 | 32,145,576 | 450,089,280 | 14.01 | 14.96 | 14.97 | 14.97 | 1.20 | 1.29 | 1.29 | 1.29 |
| South Africa | 79,583 | 6,193,929 | 61,711,520 | 9.92 | 5.18 | 11.63 | 14.73 | 0.85 | 0.45 | 1.00 | 1.27 |
| Swaziland | 2,303 | 176,912 | 807,760 | 4.57 | 2.07 | 4.24 | 6.74 | 0.39 | 0.18 | 0.36 | 0.58 |
| Togo | 603 | 51,211 | 275,643 | 5.39 | 1.63 | 3.86 | 7.95 | 0.46 | 0.14 | 0.33 | 0.68 |
| Uganda | 13,436 | 1,155,325 | 8,861,710 | 7.67 | 4.03 | 6.87 | 12.07 | 0.66 | 0.35 | 0.59 | 1.04 |
| Tanzania | 85,187 | 7,300,801 | 71,603,992 | 9.80 | 4.75 | 11.65 | 14.80 | 0.84 | 0.41 | 1.00 | 1.27 |
| Yemen | 22,994 | 1,912,083 | 28,426,742 | 14.87 | 14.93 | 14.97 | 14.97 | 1.28 | 1.28 | 1.29 | 1.29 |
| Zambia | 51,821 | 4,317,773 | 18,682,978 | 4.34 | 1.50 | 3.29 | 6.58 | 0.37 | 0.13 | 0.28 | 0.57 |
| Zimbabwe | 114,048 | 9,216,492 | 76,158,584 | 8.28 | 4.22 | 8.37 | 12.60 | 0.71 | 0.36 | 0.72 | 1.08 |
| Ilemi triangle | 2,841 | 243,683 | 3,603,087 | 14.79 | 14.92 | 14.97 | 14.97 | 1.27 | 1.28 | 1.29 | 1.29 |

**References**

1. Lundgren, K. *How will Climate Change Working Life ? Impacts of Heat on Productivity and Health*. (2015).

2. Lundgren, K., Kuklane, K., Gao, C. & Holmér, I. Effects of Heat Stress on Working Populations when Facing Climate Change. *Ind. Health* **51**, 3–15 (2013).

3. Myers, S. S. *et al.* Human health impacts of ecosystem alteration. *Proc. Natl. Acad. Sci.* **110**, 18753–18760 (2013).

4. Rosenstock, T. S. *et al.* A Planetary Health Perspective on Agroforestry in Sub-Saharan Africa. *One Earth* **1**, 330–344 (2019).

5. Elagib, N. A. & Al-Saidi, M. Balancing the benefits from the water–energy–land–food nexus through agroforestry in the Sahel. *Sci. Total Environ.* **742**, 140509 (2020).

6. Waldron, A. *et al.* Agroforestry Can Enhance Food Security While Meeting Other Sustainable Development Goals. *Trop. Conserv. Sci.* **10**, 194008291772066 (2017).

7. Chapman, M. *et al.* Large climate mitigation potential from adding trees to agricultural lands. *Glob. Chang. Biol.* **26**, 4357–4365 (2020).
